# Implementing and Maintaining a SARS-CoV-2 Exposure Notification Application for Mobile Phones: The Finnish Experience

**DOI:** 10.2196/46563

**Published:** 2023-07-13

**Authors:** Mika Pihlajamäki, Sara Wickström, Kaija Puranen, Otto Helve, Aleksi Yrttiaho, Lotta Siira

**Affiliations:** 1 Finnish Institute for Health and Welfare Helsinki Finland; 2 Gofore Oyj Tampere Finland

**Keywords:** digital proximity tracing, DPT, exposure notification application, ENA, COVID-19, Finland, digital health, mobile health, mHealth, contact tracing, user, data privacy, effectiveness, mobile app, technology, public health

## Abstract

Exposure notification applications (ENAs) or digital proximity tracing apps were used in several countries during the COVID-19 pandemic. In this viewpoint, we share our experience of implementing and running the Finnish ENA (Koronavilkku), one of the national ENAs with the highest proportion of users during the pandemic. With the aim of strengthening public trust and increasing app uptake, there was a strong prioritization of privacy and data security for the end user throughout the ENA development. This, in turn, limited the use of the app as a tool for health care professionals and deeper insight into its potential effectiveness. The ENA was designed to supplement conventional contact tracing, rather than replace it, and to serve as an early warning system and a trigger for action for the user in case of potential exposure. The predefined target of 40% uptake in the population was achieved within 3 months of the ENA launch. We consider easy-to-understand information produced together with communication experts crucial during the changing pandemic situation. This information educated people about the app as one component in mitigating the pandemic. As the pandemic and its mitigation evolved, the ENA also needed adapting and updating. A few months after its launch, Finland joined European interoperability, which allowed the ENA to share information with ENAs of other countries. We added automatic token issuing to the ENA as of mid-2021. If added earlier and more comprehensively, automatization could have more effectively saved resources in health care services and prevented overburdening contact tracing teams, while also notifying potentially exposed individuals quicker and more reliably. In the spring of 2021, the number of active apps started to gradually decline. Quarantine and testing practices for asymptomatic vaccinated individuals following exposure to the virus were eased and home tests became more common, eventually replacing laboratory testing for much of the population. Taken together, this led to decreased token issuance, which weakened the potential public health usefulness of the app. A self-service option for token issuance would likely have prolonged the lifespan of the app. The ENA was discontinued in mid-2022. Regularly conducted surveys would have helped gain timely knowledge on the use and effectiveness of the app for better responding to the changing needs during the pandemic.

## Introduction

Large-scale rollouts of digital proximity tracing (DPT) and exposure notification apps (ENAs) were undertaken hastily in several countries during the COVID-19 pandemic. It is important to gather and document the lessons learned in the process for possible similar situations in the future. In this viewpoint, we share our experiences of implementing and running the Finnish ENA (Koronavilkku). We base our account on the first-hand experiences of developing and maintaining the national ENA that were discussed at the final project workshop. We refer to feedback received from the public, health care professionals, and decision makers during the app’s lifetime.

**Figure 1:**
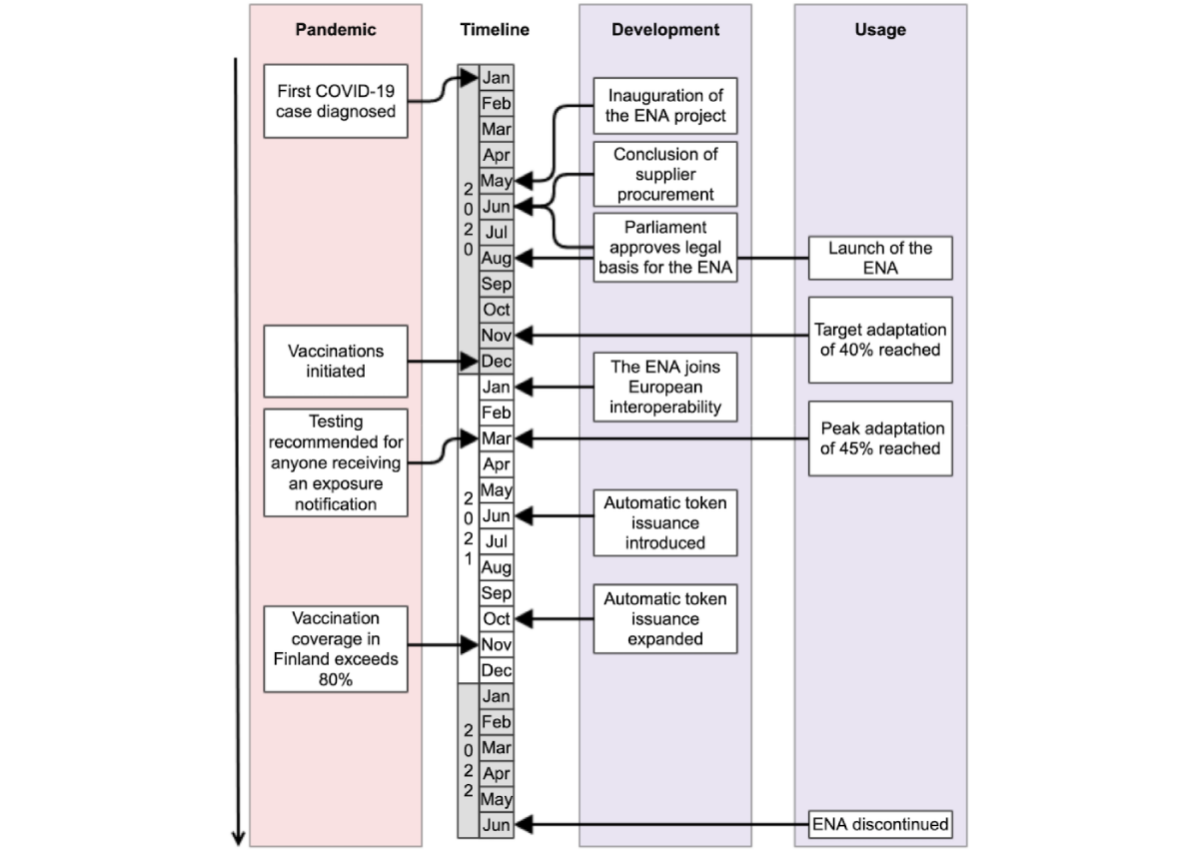
Timeline of key events in Finland during January 2020 to June 2022, related to the COVID-19 pandemic mitigation and the development and use of the Finnish COVID-19 exposure notification application (ENA).

SARS-CoV-2 started to spread in the Finnish population at the end of February 2020 [1]. Like in many countries, in mid-March 2020, the government declared a state of emergency and introduced restrictions due to the coronavirus outbreak [2]. In early April, the European Commission adopted a recommendation on the use of mobile technology to control the COVID-19 pandemic [3], and the Finnish government initiated legislation work related to a national app later that month (Figure 1). The aim of the mobile app would be to promote public health by empowering everyone to participate in breaking SARS-CoV-2 transmission chains, as part of the government’s strategy to combat the COVID-19 pandemic [4].

## Defining the Aims and Functionality of the ENA

According to the temporary law, the Finnish app would be voluntary and free for the public to use [5]. These principles are in line with findings in a review on best guidance for DPT apps [6]. The national legislation and the European guidelines defined the handling of personal data and directed the Finnish app to use a decentralized model and the Google and Apple Exposure Notification system without collecting location data [5,7]. Privacy and data security concerns have been raised by the public in studies on acceptability [8,9]. Local legislation is needed to ensure suitable and specific safeguards for privacy and data protection to gain public trust [10,11].

The Finnish app was divided into a mobile app and a backend system [12]. The mobile app calculated the exposure risk based on encounter duration, distance, and the presumed infectivity of the case. If a defined risk threshold was exceeded, the app notified the user of a potential exposure and provided further instructions. This functionality for sending and retrieving identifiers and matching them was similar in all decentralized ENAs in the European Union (EU) [13].

Based on early simulated models on potential for infection control [14,15], a high app download rate by the public was deemed more important than making the app a tool for health care professionals. In a simulation, a 56% app coverage would end the pandemic in the modelled country [14]. At that time, app coverage in other countries was up to 38% [16]. The project steering committee set the objective to 40% of the population in Finland, to have a concrete and realistic target coverage to mitigate the pandemic.

**Figure 2:**
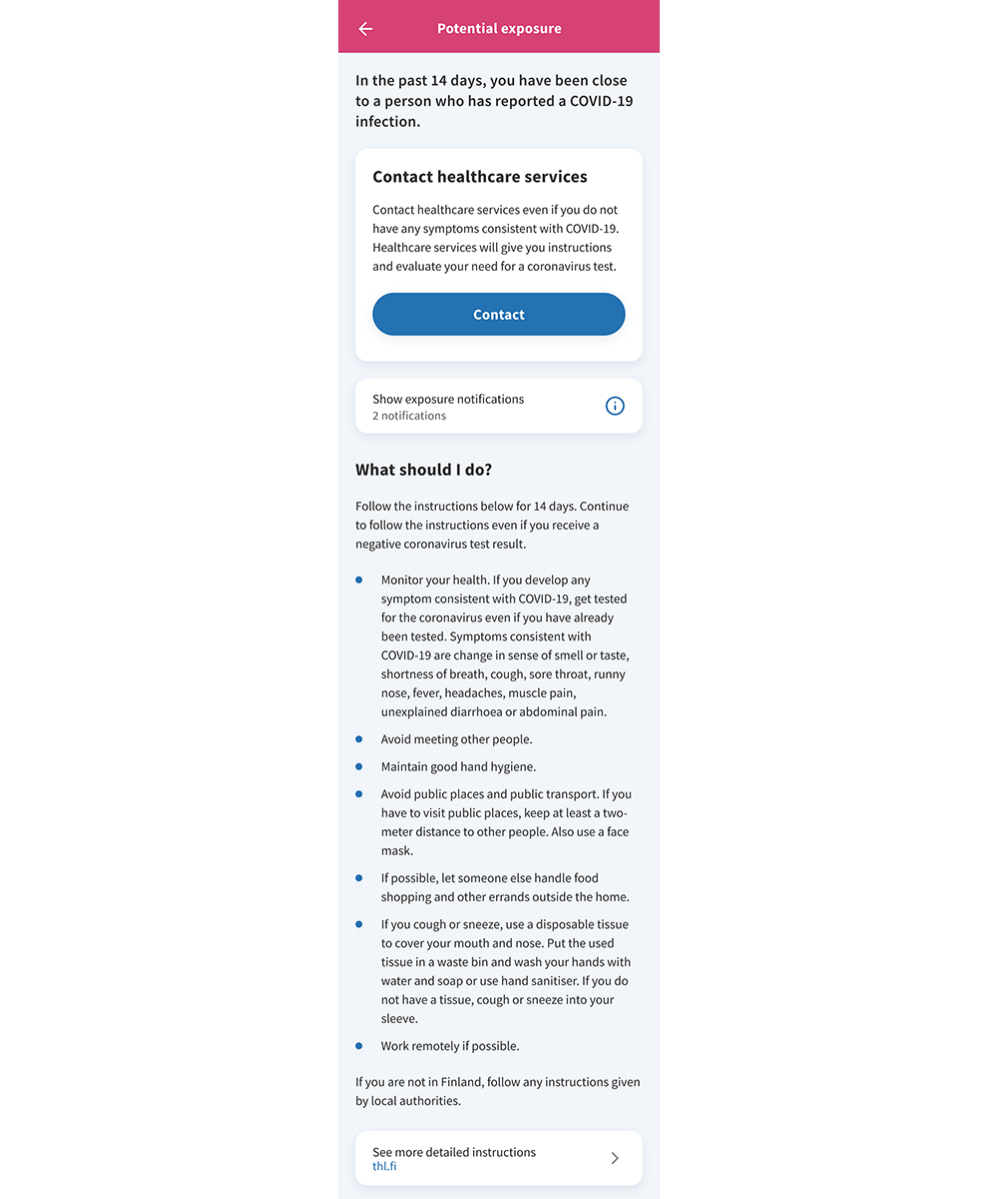
The guidance shown to users of the Finnish COVID-19 exposure notification app upon receipt of an exposure notification during March 4, 2021, to June 16, 2021. The instructions were updated in line with the national guidelines.

The app was designed to supplement conventional contact tracing, rather than replace it, and to serve as an early warning system and a trigger for end user action. The aim was that upon receiving an exposure notification, the user would change his or her behavior according to the guidance given by the app, and thus, break transmission chains (Figure 2). The instructions were kept in synchronization with the national guidelines.

Already in the planning stage, it was decided that we should emphasize privacy and maximize app uptake, which imposed limits on the development but gave a clear focus to the work. After the launch, several independent data security experts, including the National Cybersecurity Center, evaluated the app against the privacy concerns that had been discussed in the media [17,18]. We believe that the focus on privacy and data security, as well as the open communication about this aim, was instrumental in gaining public trust. The importance of upholding public trust to increase app uptake is consistent with other studies [19]. Distrust and a perceived lack of transparency can increase the belief in inadequate design, and in turn, decrease belief in app effectivity and app adoption [20]. Privacy concerns among the public are understandable, as many COVID-19–related apps required numerous rights, like access to photos, location, or microphone [21].

## Development

In most EU countries, ENAs were developed in collaboration between governmental institutions and private software developers [13]. This was also true for Finland, where establishing an ENA required several actors to join forces. The project group consisted of some 2 dozen individuals from the involved organizations. Because of the pandemic-related recommendations to work remotely, meetings were web based, and most team members met in person for the first time only at the final project workshop. One of the key lessons learned was the importance of seamless teamwork. Home organizations were less visible in the web-based meetings and discussions were informal yet structured. This fostered open communication, an excellent team spirit, a low level of hierarchy, and avoided silos, while also engaging project members and their home organizations. In addition to the core project group, nearly 200 individuals worked intermittently on different aspects of the app and related issues.

To support the project group, we set up an expert group of communicable disease physicians representing different municipalities and hospital districts. The group lacked decision-making power, but it was an important organ for keeping in touch with the health care services, where contact tracing was conducted and app tokens issued. The group was informed about the development and consulted on guidance and the calculation of risk factor parameters. The aim was to keep the parameters that defined possible exposure and recommendations in the exposure notifications in line with the national guidelines.

## Communication and Deployment

From the start, communication experts planned how to share easy-to-understand information about the ENA with the public. As identified elsewhere, end user benefits of an app and clarifications on how individual privacy will be protected are important to communicate [22]. Communication efforts included a series of public webinars during development and a marketing campaign in connection with the launch, and communication continued throughout the lifetime of the app. The responsibilities of the national COVID-19 helpline were expanded to give advice on the app and its use.

Finland approached advertising the app as part of a bigger strategy against the pandemic and started informing the public about the app at an early stage [13]. However, some other European countries included the public in the development by inviting feedback or carrying out public consultations to generate trust and increase acceptance [13]. Because of time and resource constraints, we were unable to accommodate similar public consultations.

**Figure 3:**
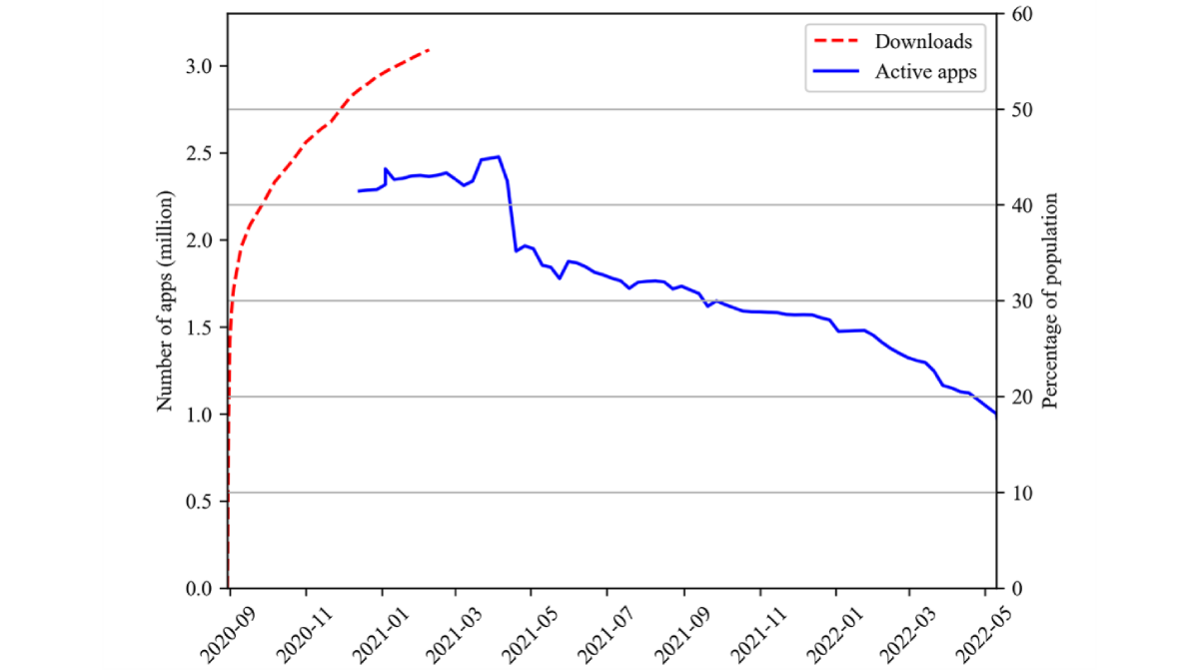
Downloads or active app counts for the Finnish SARS-CoV-2 exposure notification application (ENA) in the period of August 31, 2020, to May 10, 2022. The number of Finnish ENA downloads was collected from mobile app stores. After February 8, 2021, the number of downloaded apps was not followed, as it no longer reflected the number of active apps. Active apps were defined as apps contacting the back end at least once per 24 hours.

The Finnish ENA was published in late summer 2020 (Figure 1), simultaneously or later than that of many other countries and states [13,23]. Probably largely due to the media attention that the Finnish ENA gained, especially around the launch, the app was downloaded by 1 million users within the first 24 hours. This accounts for nearly a fifth of the Finnish population. In the next 2 weeks, the app reached 2 million users, and the objective of 40% uptake was achieved in early November 2020 (Figure 3) [24]. Over the next few months, the number of active users continued to grow, and by spring 2021, the app had become one of the national or state ENAs with the highest proportion of users [13,25].

The ENA could be used in the 2 official languages of Finland, Finnish and Swedish, and additionally in English. However, some groups were not as frequent users of the app as the general population [26], and making the app available in further languages could have allowed it to have a wider and more inclusive public health impact.

## Adaptation of the App in an Evolving Pandemic

The ENA was developed on the premise that it would be a short-term app requiring no further releases. In Finland, the legal mandates of several control and mitigation measures related to the COVID-19 pandemic were temporary. If needed, the validity of these laws was extended; during the app’s lifetime, its legal basis was extended twice. In the changing pandemic situation, several changes were required in the app.

The project received numerous requests for minor changes and ideas for implementation, and we needed an unbiased method for prioritization and approval of them. Through the use of a structured form, we identified which parts of the app, its backend system or linked information, would be affected; the time and effort required to implement the change; and the estimated public health impact. This helped us reach informed decisions on what to implement.

In the fall of 2020, the national recommendation on quarantine duration was shortened from 14 days to 10 days. In line with this, changes in the app were required, so the duration an exposure notification was visible to the user corresponded with the updated guidelines. Similar changes were implemented 3 times during the lifetime of the app. Other updates were also made, such as a modification to exposure calculations to consider all exposures over the course of a day and changes to the interface to make it more user-friendly; these changes included displaying the number of potential exposures and adding a button to manually launch exposure checking. The motivation for all these updates was to keep the ENA current, attractive, and easy to use, thereby enabling its intended public health impact.

Several European countries launched their own mobile proximity contact tracing and warning apps, and an effort to explore sharing exposure notifications across apps was pursued by the European Commission and the member states [27]. In April 2020, a common approach for contact tracing apps was defined [7], and the first European countries joined the interoperability of apps in October 2020 [28]. Finland joined in early January 2021 (Figure 1) [13].

In accordance with increased laboratory testing capacity for SARS-CoV-2 and an updated national testing strategy [29], testing was extended to asymptomatic users who received an exposure notification, if the local testing capacity allowed (Figure 1). The web-based symptom assessment tool [30] that was linked to the ENA was eventually available for 72% of the population by August 2021. This enabled a majority of the users to book a test using various digital systems or platforms accessible through the internet.

## Automatization of Token Issuance

Manual token issuance to laboratory-confirmed COVID-19 cases became an increasing burden on the contact tracing teams in the health care services as the pandemic grew. From the beginning, we recognized that automatization would have been faster, cheaper, and less demanding on health care professionals. Similar observations have also been made elsewhere [31]. We considered automatization unfeasible to implement, due to the decentralized health care and the multitude of information management systems in use. Further, early in the pandemic, there was fear and stigma surrounding contracting COVID-19 [32], and talking to a health care professional was preferred to an automatic text message communicating the positive result of a SARS-CoV-2 test.

We routinely followed the average timespan or delay from symptom onset until app token use, which seemed to depend on the workload of laboratories and contact tracing teams and increased when case numbers grew. This raised concerns about the usefulness of the app in times when fast notification of potential contacts would have been of most importance and made us revisit automatization of token issuance.

Automatic token issuance was pursued as of early 2021 in cooperation with 2 of the largest public health care operators, City of Helsinki and the Helsinki and Uusimaa hospital region, and it was taken into use in June and October 2021 (Figure 1). Tokens could be automatically issued based on digital laboratory results and patient contact information collected during the booking of a laboratory test. This made the process of allowing cases to notify potentially exposed individuals through the app quicker and more reliable, while saving resources in the health care services. Its public health impact would have been even greater if it had started early on. Notifying contacts quickly after exposure substantially affects the effect of quarantine or self-quarantine as a control measure for the spread of SARS-CoV-2 [15,33,34].

## Shutdown

The use of the app slowly dwindled over the year 2021 (Figure 3); the likely reasons were many. Vaccinated individuals were no longer required to observe full quarantine after exposure to SARS-CoV-2 and were not automatically referred to testing if asymptomatic [35]. To many vaccinated individuals, the ENA may have seemed redundant, as the guidance also applied to those who received an app exposure notification [36].

With new SARS-CoV-2 variants causing more transmission, several municipalities and hospital regions stopped issuing app tokens in late 2021 to reduce the workload of contact tracing teams. Home tests for SARS-CoV-2 became more common and gradually replaced laboratory testing for much of the population. Positive home test results could not be registered in the app, as tokens were only issued through health care providers. Implementing a self-service option for token issuance was discussed in Finland and elsewhere [37]. Although it was not feasible for this ENA, self-service options should be considered from the start for any future similar projects to safeguard the longer-term public health use of the app.

**Figure 4:**
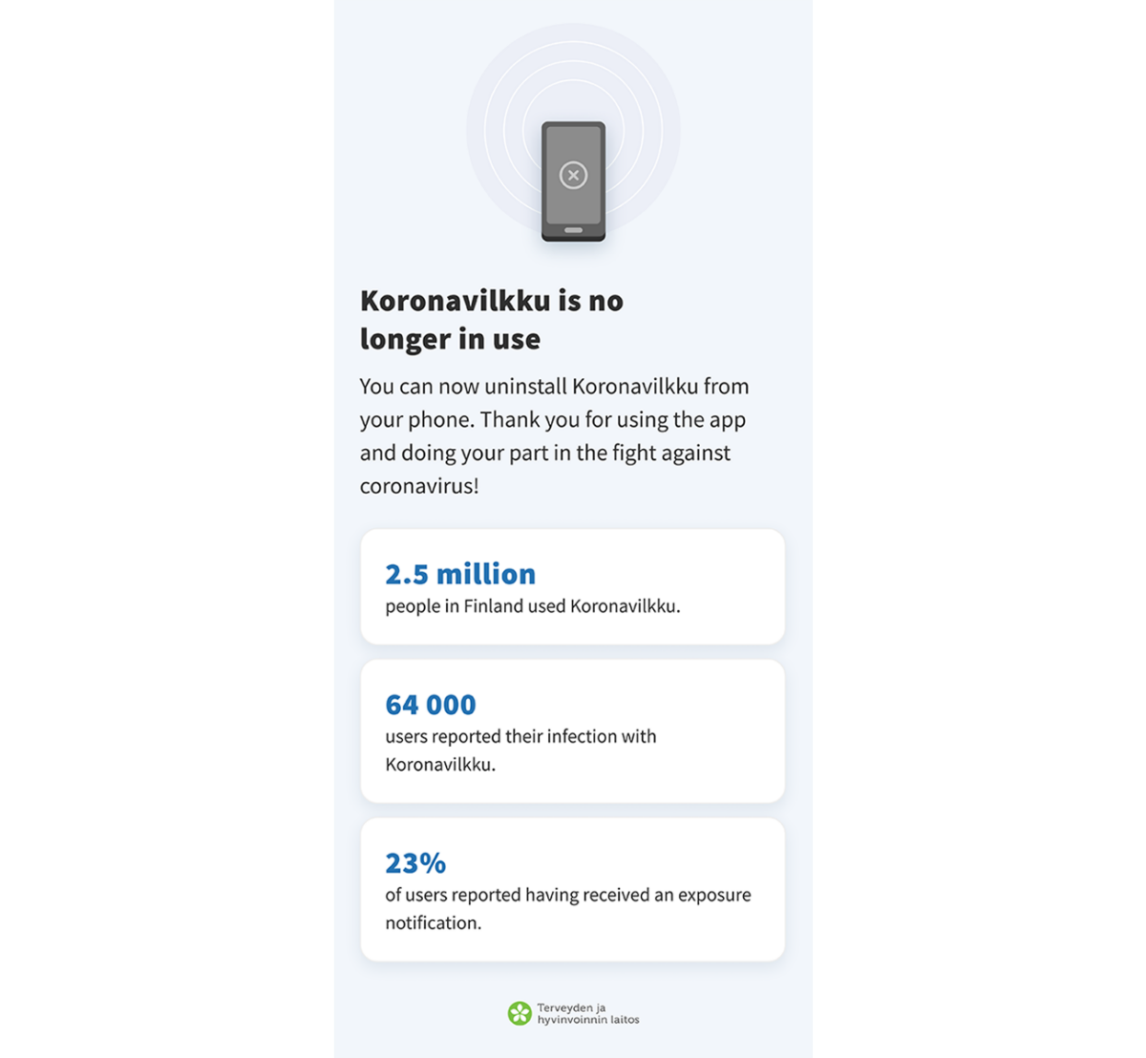
The "Thank you" screen of the Finnish SARS-CoV-2 exposure notification application—Koronavilkku. This screen was shown to users after Finland discontinued the use of the app.

We designed the Finnish ENA to gracefully retire by creating a screen that thanked the users and informed them about the shutdown (Figure 4). The ENA was discontinued on June 1, 2022 (Figure 1).

## Reflections and Recommendations

The development and launch of the Finnish ENA, Koronavilkku, illustrates how a widely used public health app, requiring new legislation, using new technology, and demanding new ways of cooperation, could be successful within a strict time frame. We consider the cornerstones of this success an excellent team spirit, low level of hierarchy within the project, open communication, avoiding silos, and a clearly defined goal. In addition, it required commitment from the project members and their home organizations.

The app was developed with a focus on the end users. Communication and marketing throughout played a key role in ensuring that potential users were familiar with the app, and we believe it contributed to the high number of downloads. Despite this, we received feedback that some health care professionals were disappointed that the app did not provide information to support their contact tracing work. In the literature, mobile apps are considered valuable tools for citizens, health care professionals, and decision makers [38], but there were difficulties in measuring and communicating these potential benefits clearly enough. In retrospect, more resources should have been allocated in the run-up to the app launch to clarify the goal to health care professionals and elaborate on the envisioned role of the app in the larger effort to mitigate the pandemic.

We believe that focusing on privacy and data security in communication and implementation strengthened public trust and, in turn, increased app uptake. However, compared to many other countries, public trust in the national government and public institutions is high in Finland [39]. Building on this trust, it may have been possible to offer the public an ENA that would have collected more information than the current interpretation of the national legislation and EU guidance allowed. In line with this, some user feedback indicated willingness to share more information with the app in exchange for receiving more information about potential exposures. This could have allowed the development of an app using the persuasive design model rather than the control design model that relies on self-monitoring. In another study [19], the persuasive design model, which uses social learning, was found to increase user intention to use the app. Enabling collection of data on exposures could have also benefited health care but would have required transparency and clear communication about the goals of data collection and its intended use. For future similar apps, we recommend enabling the collection of at least aggregated data on the number of exposure notifications generated.

Only some countries have published outcomes on their ENAs [40]. The difficulty of assessing the effectiveness of privacy-preserving DPT apps has been previously raised [23] and was also felt in relation to the Finnish ENA. The restrictions on what information we could collect through the app limited the insight into its potential effectiveness. Evidence of effectiveness may have maintained the number of users for a longer duration by swaying both health care professionals and end users to continue supporting the app. For future similar endeavors, we recommend investing in monitoring and communicating the potential benefits early in the process. If statutory limits constrain the collection of important data, regular surveys can be considered as an alternative to gain some knowledge on the use and effectiveness of such apps.

Other important lessons that we learned along the way include the fact that we did not initially foresee how many updates the app would require as the pandemic and mitigation efforts evolved. Forced updates and feeding text through a dedicated website into the app would have ensured that the guidance to users remained up-to-date, thereby enhancing the potential public health impact of the app. Another lesson learned is how a self-service option or earlier and more comprehensive automatization for token issuing for COVID-19 cases would have eased the burden on contact tracing teams and prolonged the useful lifespan of the app, while also notifying potentially exposed persons quicker and more reliably. Even with lessons learned and reflections on how the app could have been improved, the wide use of the Finnish ENA gave it potential to deliver public health and pandemic mitigation results.

## Abbreviations

DPT: digital proximity tracing
ENA: exposure notification application
EU: European Union
